# Review of the Irish Radiology Workforce: System Pressures and Future Directions

**DOI:** 10.7759/cureus.106701

**Published:** 2026-04-09

**Authors:** Riyaz Sogiawalla

**Affiliations:** 1 General Surgery, Bedfordshire Hospitals NHS Foundation Trust, Bedford, GBR

**Keywords:** artificial intelligence, health care planning, ireland, medical training, radiology training, radiology workforce, remote reporting

## Abstract

The radiology service in the Republic of Ireland operates under a well-documented workforce shortage against a backdrop of increasing service demand. This narrative review synthesizes strategic publications and insights to provide a detailed analysis of the current landscape, its challenges, and potential pathways for improvement.

Data were collated from the Faculty of Radiology, Health Service Executive, Irish Medical Council, and literature retrieved through PubMed. The aim is not to present a rigid framework but to offer a cohesive synthesis and evidence-based recommendations.

The review confirms a persistent deficit in radiologist workforce numbers, with Ireland’s density of approximately 6.5 per 100,000 population remaining substantially below the European Union (EU) median of 11.5. This shortfall is driven by increasing service demands, a training bottleneck, and modest retention of trainees. Consequences include reporting backlogs and pressure on the radiologist's wellbeing. Technological advances offer potential mitigation but cannot replace workforce expansion. The EU-REST (Radiation, Education, Staffing, and Training) project, now adopted as European Commission policy, provides a recognized benchmark for staffing.

Ireland’s radiology service stands at a critical juncture. Sustainable progress requires long-term investment in training, supervision capacity, and digital infrastructure, aligned with EU-REST benchmarks. Strategic use of technology and an expanded training program will be essential to ensure resilience and quality in radiology service delivery.

## Introduction and background

Medical imaging is a cornerstone of contemporary clinical practice, indispensable for screening programs, accurate diagnosis, procedural guidance, and monitoring of disease progression [1]. Its role influences decision-making across nearly all medical and surgical specialties. In Ireland, as in many developed nations, demand for radiological services has increased consistently year on year, propelled by demographic shifts, growing complexity of patient care, and the expansion of national health strategies [2].

However, the system responsible for delivering these critical services is facing a multitude of pressures that threaten its operational efficacy and long-term sustainability. Over the past two decades, a series of high-level reports have consistently highlighted a critical shortfall in the radiology workforce. Despite this, subsequent analyses indicate that these targets have proven challenging to meet, suggesting the presence of systemic issues that require more than incremental change.

This article is conceived as a narrative review, aiming to bring together the key findings from sequential publications and other relevant sources. The objective is not to propose a definitive framework but to collate the available evidence, trace the narrative of the workforce challenge, and offer a consolidated space for the suggestions that have emerged from this body of work. By synthesizing insights from strategic national documents, workforce surveys, and the evolving international discourse on technology in radiology, this review aims to provide an accurate assessment of the current workforce of Irish radiology. It will explore the quantitative and qualitative dimensions of the workforce shortage and examine potential future directions that could help steer the service toward a more sustainable future.

Methodology

A narrative review was conducted by systematically identifying and analyzing publications related to the Irish radiology workforce. A literature search was performed on PubMed using combinations of the terms: (“Radiology” OR “Radiologists” OR “Medical Imaging”) AND (“Workforce” OR “Staffing” OR “Staff Shortages” OR “Human Resources”) AND (“Ireland” OR “Irish” OR “Ireland healthcare system” OR “Irish medical workforce”) AND (“RVU” OR “Relative Value Units” OR “EWTD” OR “European Working Time Directive” OR “Working Time Regulations” OR “Workload” OR “Staffing Levels”). This search yielded 13 results, of which five were directly relevant to Irish radiology.

To ensure a comprehensive synthesis, this strategy was supplemented by a manual search for pivotal national strategic documents identified after reading the papers from the initial search, including the Faculty of Radiologists’ publications and Health Service Executive (HSE) and National Doctors Training and Planning (NDTP) documents. Additional sources, including longitudinal survey data from the Irish Medical Council (IMC) (via “Your Training Counts”), the Oireachtas, and recent international literature on technological advancements and workforce planning, were included due to relevance.

Inclusion and exclusion criteria are described in broad terms above, which limits formal reproducibility and allows risk-of-bias assessment. Preferred Reporting Items for Systematic Reviews and Meta-Analyses (PRISMA)-style methods were intentionally not applied, as this was deemed acceptable for a narrative policy-focused review. The intention was not to perform a systematic review with rigid inclusion criteria, but to collate the available evidence from these sequential publications to construct a narrative and consolidate evidence-based suggestions for the future.

The structure follows the evolution of the problem, from the identification of workforce shortages by the Faculty of Radiologists and HSE, first through the development of tools to measure workload activity and guide workforce planning. The discussion follows through to provide an update on the latest available numbers, comparisons with regional neighbors, and relates to recently published articles through a literature review from the Irish context. The overarching goal is to compile the literature and highlight approaches to workforce expansion that could relieve system pressures and lead to improved service delivery.

## Review

Quantifying the workforce deficit

Accurate assessment of workforce adequacy depends on first establishing a reliable method of quantifying workload. Recognizing this, the Faculty of Radiologists undertook a national workload survey in 2009, culminating in the publication of its 2011 Guidance on Radiologist Workload Figures and a companion editorial in European Radiology. This landmark initiative introduced a relative value unit (RVU) model adapted from the Royal Australian and New Zealand College of Radiologists (RANZCR) to objectively measure radiologist output across modalities and hospital types [3].

The survey analyzed returns from 28 of 38 public hospital radiology departments, assigning RVU weights to various study types and quantifying time spent on “non-countable” activities such as multidisciplinary meeting (MDM) preparation, teaching, interventional work, and administration. The mean workload was 57,659 RVU per whole-time equivalent (WTE), substantially higher than international benchmarks then reported by RANZCR (40,000-45,000 RVU/WTE). Findings also revealed that Irish radiologists devoted approximately one-third of their working time (mean 32%) to such non-interpretive duties.

The 2011 framework thus established the first national quantitative benchmark for radiologist activity, marking a shift from anecdotal to data-driven workforce planning. It also underscored that a significant portion of radiologists' work was invisible to traditional activity metrics such as reporting, highlighting the importance of incorporating non-interpretive functions in future workforce assessments.

Subsequent Faculty revisions in 2020 and 2024 refined this methodology, integrating new data on MDM preparation, outside-film consultations, and procedural work [4,5]. These later documents retained the RVU framework introduced in 2011 but added stronger guidance on balancing productivity with professional wellbeing and explicitly linked excessive workload to burnout and diagnostic error risk. Collectively, these reports provided a structured, evidence-based foundation.

With a framework for workload measurement established, the next step is to review the scale of the workforce deficit. Quantification requires linking measured radiological activity with population-based benchmarks and available consultant capacity. This step marked an important transition, from describing workload pressures at the departmental level to expressing the national shortfall in measurable terms.

The 2016 Faculty of Radiologists Strategic Review quantified the shortfall, reporting only five radiologists per 100,000 population, below the European average of eight and far behind France at 11 [6]. It recommended doubling consultant numbers from 267 to 650 by 2026 and expanding training capacity accordingly.

The most comprehensive national analysis to date, published by the HSE and NDTP in 2025, reported 352 consultant radiologists (~6.5 per 100,000 population) and concluded that the primary constraint on growth was training program capacity rather than recruitment [7]. Two workforce demand scenarios were modeled, projecting a requirement of 770-1,270 WTE radiologists by 2040. The report proposed increasing annual HST intake from 39 to 75 by 2028, contingent on funding and supervision capacity.

To fully appreciate the Irish situation, it is useful to place it within the broader European context. The EU-REST (European Union-Radiation, Education, Staffing, and Training) study, commissioned and then published by the EU Commission, established guidelines for education, training, and staffing for all staff working with ionizing radiation across the EU 27 states. A series of papers published in Insights into Imaging to disseminate the information specific to radiologists from the EU-REST found that the EU radiologist density median is approximately 11.5 per 100,000 population, with wide variation from five to over 27 per 100,000, and Ireland’s figure of 6.5 per 100,000 sits well below the median [8-10].

Beyond identifying numeric disparities, the EU-REST framework introduced a structured, evidence-based method to model staffing needs. It proposed a "basic unit" of one hour of system use as a universal metric to calculate radiologist demand across modalities and hospital types, allowing adjustment for academic settings, trainee supervision, and evolving technologies such as artificial (AI) intelligence. This approach enables workforce projections to remain flexible as clinical practice evolves, for example, as interventional procedures shift from diagnostic to therapeutic roles, or as AI tools alter time allocation per case [11].

Most importantly, the NDTP 2025 document explicitly references the EU-REST project, aligning Ireland’s projections with the European Commission’s newly adopted staffing and education benchmarks. Ireland’s adoption of these metrics through the NDTP framework transforms national workforce targets into policy-anchored objectives. Achieving parity with EU-REST norms will require sustained expansion of training posts, improved data collection, and a stable pipeline of consultant appointments.

Underlying causes of the workforce deficit

Training Capacity and Supervision

The apprenticeship model of radiology training demands close supervision by consultants, limiting scalability. Expansion requires sufficient trainers, reporting workstations, and physical space, resources already constrained in many hospitals. The NDTP review advocates expansion of HST intake; however, expansion will depend on dedicated funding streams and collaboration between the Faculty and training hospitals.

Health Information Technology

Underinvestment in health infrastructure has been identified as a barrier to workforce efficiency. In 2025, an Oireachtas Research Matters report noted that Ireland was the lowest-ranked EU country for citizen access to eHealth records, and only five of 47 public hospitals had their own electronic health records (EHRs) [12]. The HSE’s Digital Health Strategic Implementation Roadmap (2024) identifies diagnostic services integration, including the National Integrated Medical Imaging System (NIMIS), as key to improved workflow [13]. Poor digital infrastructure increases the administrative burden on radiologists and contributes to non-reporting tasks. Moreover, fragmented information systems lead to duplication of effort, repeated investigations, and inefficiencies in clinical communication. This not only compounds workload pressures but also amplifies the perception of workforce shortage, as time and expertise are diverted away from core diagnostic activity.

Retention and Outward Mobility

Retention of trainees within Ireland remains modest, with around 61% of trainees practicing domestically post-training [7]. Fellowship training abroad is an established component of Irish medical education, allowing exposure to advanced subspecialties. The issue, therefore, is not outward migration per se but the conditions influencing whether fellows return. Surveys (e.g., “Your Training Counts”) show that professional support, manageable workloads, and structured career progression are strong incentives for return [14-16].

Systemic consequences

Reporting Backlogs and Diagnostic Delays

Persistent understaffing translates directly into reporting delays. National Radiology Quality Improvement Programme (NRQI) national data show fewer than half of sites meet the Faculty benchmark of 90% of reports authorized within designated times (10 days for outpatient, 12 h for emergency CT/MRI) [2]. Smaller hospitals and those with fewer consultants are also disproportionately affected.

Radiologist's Wellbeing and Diagnostic Performance

The Faculty’s workload guidance documents explicitly state that unmanageable workloads and unrealistic deadlines are key contributors to burnout [3-5]. Errors and discrepancies in image interpretation arise not from individual failings but from cumulative pressures within the working environment [17]. Perceptual and cognitive errors, often exacerbated by fatigue, time pressure, and high reporting volumes, remain an inherent aspect of imaging practice. When departments operate at or beyond capacity, the margin for diagnostic accuracy narrows, and opportunities for peer review, reflection, and quality improvement become constrained.

A system that prioritizes protected reporting time, collaborative review, and open discussion of discrepancies, focusing on shared learning, is therefore essential to maintaining both diagnostic quality and staff wellbeing.

Pathways for alleviation: technology and innovation

Remote and Hybrid Reporting

Experience from the COVID-19 era confirmed that remote reporting can enhance flexibility and resilience. A 2020 pilot at Imperial College NHS Trust demonstrated a 140% increase in consultant output using a secure home workstation model and a 40% reduction in the time taken to sign registrar reports [18]. For Ireland, where consultant distribution is uneven, structured hybrid models could help balance workload nationally. Implementation requires investment in secure broadband infrastructure, diagnostic-grade monitors, and integration with NIMIS.

Artificial Intelligence

AI applications have shown potential in prioritization and triage, but have a limited impact on overall productivity. A UK study trained convolutional neural networks to classify chest radiographs into critical, urgent, nonurgent, or normal, achieving 95% specificity and 71% sensitivity; reporting delay for critical findings reduced from 11.2 to 2.7 days [19]. The EU AI Act (Regulation 2024/1689) (June 2024) and the European Society of Radiology (ESR) 2025 guidance focus on transparency, data quality, and human oversight [20,21]. For Ireland, success will depend on rigorous local validation, interoperability with existing systems, and national governance structures.

Training Pathway Innovation

The most fundamental intervention to address the workforce shortage is the expansion of training capacity. Models implemented elsewhere include regional training pathways and structured radiology academies. For instance, the RANZCR regional and rural training pathway encourages doctors to train in rural locations and limits time in metropolitan hospitals to one-third of the program, thereby supporting recruitment and retention in underserved areas [22,23]. In the UK, radiology academies provide multidisciplinary training environments for the wider imaging workforce [24]. However, the suitability of these models for Ireland has not been specifically evaluated. Reviewing such models, or developing new ones tailored to the Irish context, could enhance training capacity, strengthen rural service provision, and alleviate existing training bottlenecks.

**Figure 1:**
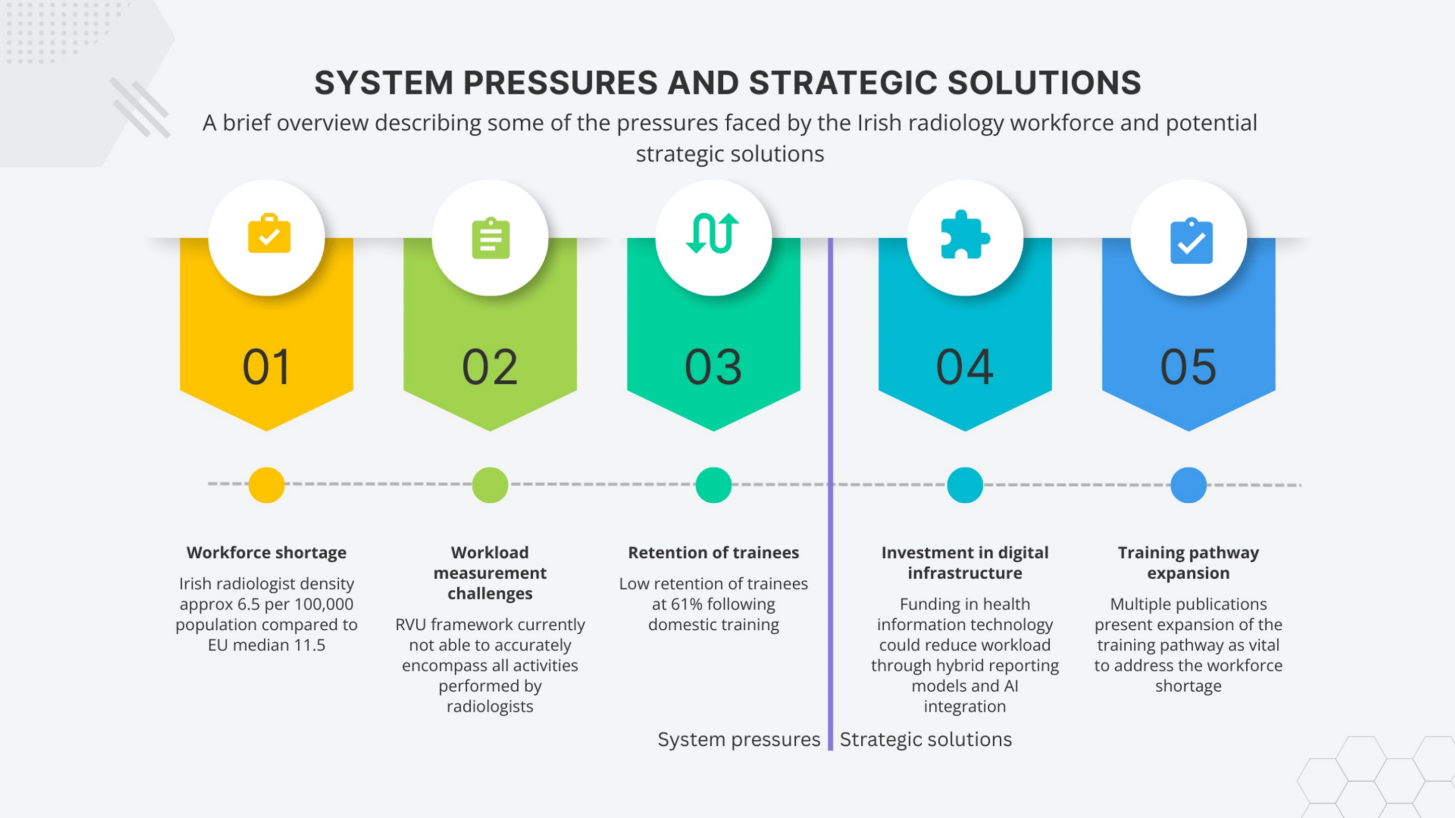
Conceptual graphic illustrating system pressures and proposed strategic solutions for Ireland’s radiology workforce. Image Credit: Author created using Canva (Canva Pty Ltd., Sydney, Australia).

Figure 1 below summarizes that the system pressures, along with increased workload, have amplified workforce strain, and includes discussed solutions that offer a pathway to sustainable reform.

Limitations

This review focuses primarily on consultant radiologists and trainees and does not explore the significant contribution of the wider imaging workforce, radiographers, sonographers, physicists, and radiology nurses, who are integral to service delivery. The review also has not addressed equipment age, availability, or consultant retirement projections and has relied heavily on policy documents. Future work to incorporate these elements would provide a fuller assessment of Ireland’s imaging workforce shortage.

Growth in training capacity is essential, but must be balanced with maintained training standards and robust supervisor support. Investment in digital infrastructure is also pivotal not only to reduce workload through improved information sharing but to enable AI and remote working tools effectively. A coordinated, multi-layered strategy is essential: combining workforce expansion, adopting advanced technology, and a culture change toward continuous improvement to secure a sustainable, high-quality radiology service for the future.

## Conclusions

In compiling key insights from over a decade of strategic reporting and international comparisons, the evidence presents a consistent picture: Ireland’s radiology service is under pressure from a longstanding workforce shortage, a constrained training pathway, and issues with retention, exacerbated by rising demand and expanding non-interpretive duties for radiologists. Targeted efforts by policymakers and the training body are needed to lead a transformation to improve Ireland’s radiology services.
